# Older Adults Making End of Life Decisions: An Application of Roy's Adaptation Model

**DOI:** 10.1155/2013/470812

**Published:** 2013-12-26

**Authors:** Weihua Zhang

**Affiliations:** Nell Hodgson Woodruff School of Nursing Emory University, 1520 Clifton Road, Atlanta, GA 30322-4207, USA

## Abstract

*Purpose*. The purpose of this study was to identify variables that influenced completion of advanced directives in the context of adaptation from national data in older adults. Knowledge gained from this study would help us identify factors that might influence end of life discussions and shed light on strategies on effective communication on advance care planning. *Design and Method*. A model-testing design and path analysis were used to examine secondary data from 938 participants. Items were extracted from the data set to correspond to variables for this study. Scales were constructed and reliabilities were tested. *Results*. The final path model showed that physical impairment, self-rated health, continuing to work, and family structure had direct and indirect effects on completion of advanced directives. Five percent of the variance was accounted for by the path analysis. *Conclusion*. The variance accounted for by the model was small. This could have been due to the use of secondary data and limitations imposed for measurement. However, health care providers and families should explore patient's perception of self-health as well as their family and work situation in order to strategize a motivational discussion on advance directive or end of life care planning.

## 1. Introduction

Those aged 65 and older represented 35.9 million or 12.3% of the US population in 2003, and this number will reach 19% by the year 2030 [1]. The great majority of deaths (80–85%) occur in this population, and most die from chronic conditions such as heart failure, cancer, obstructive pulmonary disease, diabetes, Alzheimer's disease, and renal failure [2]. The Institute of Medicine (IOM) noted that while technology continues to increase life expectancy, the quality of life of dying patients has not kept the same pace [3]. Studies indicate that patients' families are still dissatisfied with end of life care [4–7]. Advance directives (ADs) were seen as a way for individuals to decide on and communicate their end of life care wishes to those who care for them to ensure, more in a legal term, that individuals' wishes for end of life care are followed. ADs include durable power of attorney and a living will. However, more than 2 decades after passage of the 1991 Patient Self-Determination Act (PSDA) requiring AD discussion on admission to health care institutions, the rate of completion of ADs remains at approximately 29% of the population [8]. Advance care planning is starting to replace the term “advance directives” to make its slow transitioning from a legal model to a tool to enhance communication [8]. However, factors learned from those who have completed the advance directives could help us identify factors that might influence end of life discussions [9–12] and shed lights on strategies on effective communication on advance care planning. The intent of this study was to identify and analyze factors influencing AD completion in an older population.

## 2. Literature Review

The literature on outcomes of end of life communication is focused on AD completion for this study. Those studies explore the influence of demographic factors such as age, gender, family relationships, and health issues on the completion of ADs.

Studies indicate that older white women with more education who perceive themselves to be in poor health are more likely to have signed an AD [13–16]. Douglas and Brown [17] also reported that women were more likely than men to have completed ADs. However, Cooper and colleagues [18] found that gender was not related to AD completion.

Race may also be related to AD completion. Study reported that African-Americans were significantly more likely to choose aggressive life-saving interventions and less likely than Caucasians and Asians to complete ADs [19, 20]. Eleazer and associates also noted that Hispanics were more likely to refuse to complete any form of ADs [19]. In support of Eleazer et al.'s finding, Degenholtz et al. [21] also found that African-Americans and Hispanics were less likely than Caucasians to have living wills. However, Morrison and associates [22] reported that race was not significantly related to the signing of a durable power of attorney. Nolan et al. reported that gender, race, and health status are not significantly related to AD completion [23].

Investigators in two studies found that those with higher incomes, health insurance, and more trust in the medical system were more likely to complete ADs [24, 25]. Findings from other studies note that those with less education were significantly less likely to sign ADs and more likely to desire life-sustaining treatment [26, 27]. It is evident that studies examining demographic variables have diverse findings. Overall, age, income, health insurance, and more trust in medical system are positively related to AD completion. However, age-related variables, such as poor health and more physical impairment, could complicate the relationship between age and AD completion.

In Gerald's [14] study, neither diagnosis nor marital status was significantly related to AD completion. However, more widows or widowers had signed an AD than those who were married, single, or divorced. Colenda et al. [28] reported that those who lived close to their children and those who with large social networks had lower AD completion rates. Studies indicated that those had close relationships with their families [29] or have family members living nearby [30] tended to desire less aggressive life-saving interventions than those who did not have close relationships.

Although the completion rate for ADs is about 15% to 25% in adults [10], hospitalization or perception of poor self-health may precipitate completion of ADs. Studies indicate that those who had a recent hospitalization completed ADs more than those who had not been hospitalized [25, 31]. McAuley and associates reported that after a 12-month nursing home admission, AD completion rates increased by 18% from the baseline [32]. Morrison et al. [22] and Salmond and David [10] found that poorer self-reported health status was significantly related to AD completion. Hospital admission, suffering an illness, and decline of health status could prompt more AD completion. Being informed about ADS at admission during a health care crisis could also contribute to more completion upon hospitalization.

The relationships among age, gender, race, education level, family structure, and health factors to AD completion have been studied separately. Variables that influence decision making on AD completion can be analyzed in a contextual model. How those external and internal factors (stimuli), modes of adaption, and decision-making factors working together to influence AD completion has yet to be examined. The purpose of this study is to apply Roy's Adaptation Model to analyze the influence of demographic, family structure, and health status variables on AD completion.

**Figure 1:**
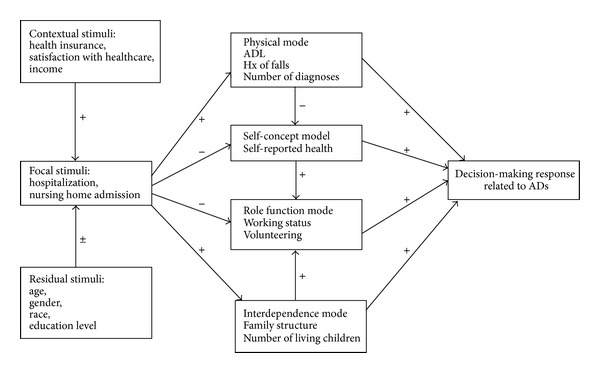
Application of the RAM to AD Completion.

Roy's Adaptation Model (RAM) [33] provides a framework for identifying variables that influence individuals' responses to stressful situations (Figure 1).

According to Roy, individuals mobilize all possible ways to adapt to a stressful situation. An individual's ability to adapt to situations varies depending on the nature of the stimuli confronting the person. Stimuli are focal, contextual, or residual. Focal stimuli are influenced by contextual and residual stimuli. Contextual stimuli are internal or external factors that influence how the individual reacts to the focal stimuli but do not require direct and immediate attention or energy. Residual stimuli are factors affecting the person with an uncertain effect.

Focal stimuli exert a direct and immediate effect on the individual and become the focus of attention for the person. The person mobilizes control processes which consist of regulator and cognator processes [34]. Neural, chemical, and endocrine coping processes (physiological) are termed regulator processes, while cognator processes result from cognitive-emotive channels. The person spends energy to mobilize four adaptive modes: physical, self-concept, rolefunction, and interdependence mode in dealing with the focal stimuli in order to maintain or restore adaptation through stressful event [33]. The assumption of using this theory is that each individual under any stressful situation would attempt to balance the interrelationship of physical, self-concept, role function, and interdependence mode in order to maintain or restore adaptation.

As shown in Figure 1, incorporating variables identified by the literature and placing them in the RAM provided the framework for this study. The focal stimulus was the number of hospitalization and nursing home admissions. Contextual stimuli for this study were health insurance, household income, and satisfaction with health care because those variables have shown relevance to AD completion. The residual stimuli were age, gender, race, and education level because those variables' relationships to the AD completion are not consistent. The contextual and residual stimuli were expected to have direct effects on focal stimuli (having a stressful event with AD completion status inquiry prompted by each hospitalization is operationalized as number of hospital and nursing home admissions) and indirect effects on the adaptive modes (physical, self-concept, role function, and interdependence mode) and, finally, on the decision-making responses (having signed ADs or not). Adaptive modes were expected to have direct or indirect effects on signing ADs. The regulator and cognator control processes were not measured in this study.

## 3. Design and Methods

A model-testing design based on the RAM and path analysis was selected for this study. This allowed examination of the predictive effects from one concept to the next one as identified in Figure 1. Secondary data containing information on AD completion were used for the study.

### 3.1. Sample

Noninstitutionalized US citizens aged 55 or older participated in the 1984 Longitudinal Study of Aging (LSOA) which included the 1984 National Health Interview Survey, the 1984 Health Insurance Supplement, and the 1984 baseline Supplement on Aging (SOA). The LSOA was followed by three follow-up interviews. The first follow-up interview was the 1994 Second Supplement on Aging (SOA II), which included those who had participated in the National Health Interview Survey and added a new cohort of those over the age of 70. The second follow-up interview (1997-1998) included data on those who had died since 1994. Information on those who had died (the decedent interview file) was collected from family members of the deceased. Data from the 1994 SOA II and the 1998 decedent file were extracted and merged for this study since these data are termed relevant to AD completion and also contain baseline information on demographics, physical function, working status, and family relationships. The sample consisted of the 938 participants in the merged dataset.

### 3.2. Instruments

Instruments were constructed from items identified as appropriate to the concepts of interest. The reliability of each constructed instrument with more than one item was then tested [35]. Instruments with less than a Cronbach's alpha of 0.70 were revised.

*Focal, Contextual, and Residual Stimuli. *The number of admissions to the hospital and the number of nursing home admissions were originally designed to measure the focal stimulus. These were selected as the focal stimuli because they would reflect the number of times persons were asked about AD completion status. The low Cronbach's alpha for these two items (0.31) and the fact that 732 participants had missing data on the number of nursing home admissions led to the decision that only the number of hospital admissions would be used to measure the focal stimulus.

The contextual stimuli were defined as health insurance status, household income, and satisfaction with health care. Health insurance and income were selected because they are closely related and affect both the quality and extent of health care delivery. Satisfaction with health care was chosen as an indicator of trust in the correctness of the healthcare provider's decisions and advice, and higher scores meant greater satisfaction. Age, gender, race, and education were considered as residual stimuli. 

*The Adaptive Modes. *The physical mode was measured by summing up the activities of daily living (ADLs), the number of falls, and the total number of diseases. Higher scores indicated more physical impairment. Reliability for the constructed physical mode scale was 0.72. The self-concept mode was operationalized as self-rated health status. Working status and/or doing volunteer work were measuring role function. Working or engaging in volunteer activities was assumed to provide more opportunities for social engagement. Living arrangement and size of the family were chosen to measure the interdependence mode. The higher the score, the more emotional support provided to the participants. This constructed scale had a Cronbach's alpha of 0.75. 

*Adaptive Response.* Having a completed AD indicates either having had an end of life discussion with family or a health provider or having given at least some thoughts to end of life care planning. The adaptive response for this study was having signed or not signed either a living will, a durable power of attorney, or both.

## 4. Results

### 4.1. Sample Characteristics

As shown in Table 1, the mean age of the 938 participants was 80 years old. Of the 938 participants, about half were women and most were Caucasian. More than half had at least a high school education. About half were married and lived with their spouses, and about a third lived alone. Roughly half had household incomes below $20,000 in 1994, and 256 participants did not report income. Virtually all had Medicare, and about a fourth had some other type of health insurance in addition to Medicare.

### 4.2. Descriptive Statistics for Variables in the Model

All variables except race and gender were continuous and normally distributed.  Table 2 shows the mean, standard deviation, possible range, and obtained range for each variable or constructed scale. A total of 5% had never been hospitalized and 37% had been hospitalized once, with the remainder being two or more times hospitalized from 1994 to 1997. Only 22% of the sample responded to the item on number of nursing home admissions. Of these, 80% had one nursing home admission, and 16% had more than one nursing home admission. Most (93%) ranked satisfaction with their health care provider as good or excellent. A total of 78% had a high school education or less. The majority (60%) scored 6 or less on activities of daily living 6 months prior to death, and 53% reported no falls for the four-year period. About 65% reported having been diagnosed with three or more diseases. More than half (54%) considered themselves in good or excellent health. Only 4.5% were working, and 8% were doing some volunteer work. About half (51%) lived with their spouses. A living will had been completed by 52% of the participants.

### 4.3. Path Analyses

*Procedures.* The assumptions of normal distribution, homoscedasticity, and linear relationships were met for analyses using multiple regressions. Six endogenous variables (the focal stimulus; the physical, self-concept, rolefunction, and interdependence modes; and AD completion) along with seven exogenous variables (insurance, household income, satisfaction with healthcare, age, gender, race, and education level) were included in the initial model. First, bivariate correlations were examined to give a sense of which variables might be important. Next, preliminary multiple regressions analyses were done with each of the dependent endogenous variables in turn as predicted by the theoretical model. Using results from the preliminary multiple regressions, spurious relationships were identified and eliminated. Another set of multiple regression analyses were performed with only the significant independent variables as identified in the preliminary regression analyses entered. The results from that set of analyses provided the path coefficients for the final model.

**Figure 2:**
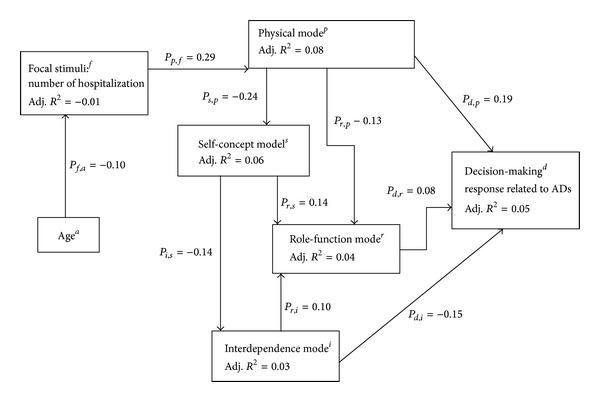
Final path model for AD Completion.

*Path Analysis Results.* From the left of the theoretical framework to the right (Figure 2), Pearson's correlation coefficients were calculated to assess bivariate relationships between the independent variables (contextual and residual stimuli) and the dependent variable (focal stimulus). Only age (*r* = −0.09, *P* = .01) was statistically significantly related to the focal stimulus. Munro [36] stated that *r*-values less than 0.49 were low and an *r*-value less than 0.25 indicates that little if any correlation exists.

Bivariate relationships between the focal stimulus and the four adaptive modes were examined next. These showed that the focal stimulus was significantly related to the physical mode (*r* = 0.29, *P* = 0.00) and the self-concept mode (*r* = −0.10, *P* = 0.01). The physical mode was also significantly related to the self-concept mode (*r* = −0.24, *P* = 0.00) and the rolefunction mode (*r* = −0.16, *P* = 0.00). The self-concept mode was significantly related to both the role function mode (*r* = 0.16, *P* = 0.00) and the interdependence mode (*r* = −0.14, *P* = 0.00).

The bivariate relationships of the four adaptive modes to AD completion indicated that only the physical mode (*r* = 0.17, *P* = 0.00) and the interdependence mode (*r* = −0.15, *P* = 0.00) were significantly related to AD completion.

The preliminary multiple regression analysis, with number of hospitalizations (the focal stimulus) as the dependent variable, showed that only age was a significant predictor (*β* = −0.10, *P* = 0.01).

The next set of multiple regressions assessed the predictive (independent) effect of the focal stimulus on each of the four adaptive modes and then the paths from one mode to the next mode. These results showed that the focal stimulus significantly predicted only the physical mode (*β* = 0.29, *P* = 0.00). The physical mode had a predictive inverse relationship with the self-concept mode (*β* = −0.24, *P* = 0.00). The role function mode was significantly predicted by the physical (*β* = −0.13, *P* = 0.00), self-concept (*β* = 0.14, *P* = 0.00), and interdependence modes (*β* = 0.10, *P* = 0.00). The results suggested a potentially significant path between the self-concept and interdependence modes, thus an additional multiple regression was done. Self-concept was identified as a significant predictor to the interdependence mode (*β* = −0.14, *P* = 0.00).

The last set of multiple regressions assessed the predictive effects of the four modes on the decision-making response. Three of the four modes were significant: they are the physical mode (*β* = 0.19, *P* = 0.00), role function mode (*β* = 0.08, *P* = 0.02), and the interdependence mode (*β* = −0.15, *P* = 0.00).

The final path model is presented in Figure 2. The physical, role, and interdependence modes jointly explained 5% of the variance in AD completion (adjusted *R* ^2^ = 0.05). The physical mode explained 6% of the variance in the self-concept mode. The physical and self-concept modes together explained 4% of the variance in the rolefunction mode. The self-concept and rolefunction modes explained 3% of the variance in the interdependence mode. The focal stimulus explained 7% of the variance in the physical mode, and age explained 1% of the variance in the focal stimulus. These findings indicate that the variance explained by application of the RAM to the study variables was not very strong. However, the soundness of the final path model was confirmed by decomposition of the bivariate correlations [37].

## 5. Discussion and Conclusion

The RAM helped to put the concepts of the interest of this study into perspective and guided the analysis of how each variable influenced other variables. However, physical impairment, remaining working, and family structure explained only a small percent of the variance in decision making on AD completion. It might be that the constructed instruments did not completely operationalize the concepts which is a disadvantage to using secondary data sets [38]. Or it can also suggest the complexity of the AD completion process. Conducting a study with instruments to fully operationalize the concepts would be helpful or an AD completion process could be a decision-making process beyond reasonable decision making.

There was no significant path from contextual stimuli and residual stimuli to the focal stimuli except for age, and age had a very small path coefficient. Contrary to the RAM, this suggests a direct path between the contextual and residual stimuli to the adaptive response. This also suggests that a possible theoretical framework modification for future research is needed. This same proposition was pointed out by a study done by Robinson [39] where a direct path from the contextual stimuli to the adaptive response was proposed.

This study supports other reports where hospitalization precipitates AD completion but only where there are decrements in physical function as well. The greater the physical impairment, the more the were completed. Participants in this study were relatively older and noninstitutionalized, so the AD completion rate of more than 50% suggests that ADs are more acceptable to this population. It is also logical to deduce that hospitalization reflects more physical impairment, and more physical impairment should make individuals more aware of the issues related to end of life care and more receptive to AD information. The path model showed that the physical mode had the greatest positive influence on decision making on ADs. This is supported by the literature [40, 41]. The paths also indicated that those who stayed connected with society were more likely to have AD completed. It is possible that those who were connected with society through work had opportunities to discuss a variety of health concerns with others, including end of life care. It may also be attributed to those who were still working or doing volunteer work wanted to maintain their independence through making their own end of life decisions. Those living in close proximity to their family were less likely to complete an AD. Such individuals may expect family members to decide on end of life care under the assumption that family members know what they would want rather than complete a legal document.

It is also worth noting that those who had discussion on ADs with their family and healthcare providers did not necessarily complete ADs. Decision making on AD completion is an ensemble of processes and cannot be predicted by a single variable or a single process. Recent literature has focused on effectiveness of discussion on end of life care planning [8]. This study provided some variables (such as physical impairment, self-rated health status, family closeness, and social involvement) that could help to design a motivational interview model for advance care planning discussion [42]. Healthcare workers should provide information in a sensitive way, without causing undue stress. Advance care planning discussions in settings other than hospitals might be more natural and less stressful. Recent study also supports this model of care and stated that early discussion should emphasize managing symptoms, strengthening coping, and cultivating illness understanding [43]. Education programs should encourage patients and families to actively participate in this discussion as early as possible. Those who received health care provider's personal request on initiating advance care planning have high completion rates on advance directives [44]. Health care workers in any health care setting should assess each individual's physical function, personal perception of the illness, family, and social factors and determine the right approach to promote adaptation. Further studies are needed to develop reliable instruments to study variables that promote adaptation and improve quality of life at the end of life by introducing a motivational discussion actively participated by patients and family.

## Figures and Tables

**Table 1:** Demographic characteristics (*N* = 938).

Characteristic	*N*	(Percent)
Age (range = 70–99, M = 80, SD = 6.84)	938	
Gender		
Male	460	(49.0)
Female	478	(51.0)
Race		
White	828	(88.3)
Black	93	(9.9)
Others	17	(1.8)
Education level		
Less than 12 years	413	(44)
12 or more years	497	(53)
Unknown	28	(3)
Marital status and living arrangement		
Married, living with spouse	481	(51.3)
Widowed	357	(38.0)
Living alone	295	(31.4)
Household income		
Less than 20,000	396	(58.1)
More than 20,000	286	(41.9)
Health insurance		
No insurance	4	(0.4)
Medicaid only	3	(0.3)
Medicare only	722	(76.9)
Private insurance or more than one	209	(22.2)

**Table 2:** Descriptive statistics for variables in the model.

Variable	M	(SD)	Possible range	Obtained range
Focal stimulus				
Number of hospitalizations	2.30	(2.00)	NA	0–24
Contextual stimuli				
Health insurance	2.34	(0.80)	0–8	0–6
Household income	5.22	(2.15)	1–9	1–9
Satisfaction with HC	3.45	(0.64)	1–4	1–4
Residual stimuli				
Education	3.60	(1.40)	1–7	1–7
Physical mode				
ADL score	6.07	(3.48)	3–15	3–15
Fall score	1.78	(0.89)	1–3	1–3
No. of diseases	3.19	(1.53)	0–10	0–8
Total	**10.89**	**(4.42)**	**4**–**28**	**4**–**25**
Self-concept mode	2.62	(1.17)	1–5	1–5
Role function mode	1.12	(0.32)	1-2	1-2
Interdependence mode				
Family structure	6.92	(3.62)	NA	2–7
AD completion	1.57	(0.49)	1-2	1-2
